# In-plane chemical pressure essential for superconductivity in BiCh_2_-based (Ch: S, Se) layered structure

**DOI:** 10.1038/srep14968

**Published:** 2015-10-08

**Authors:** Yoshikazu Mizuguchi, Akira Miura, Joe Kajitani, Takafumi Hiroi, Osuke Miura, Kiyoharu Tadanaga, Nobuhiro Kumada, Eisuke Magome, Chikako Moriyoshi, Yoshihiro Kuroiwa

**Affiliations:** 1 Department of Electrical and Electronic Engineering, Tokyo Metropolitan University, 1-1, Minami-osawa, Hachioji 192-0397, Japan; 2 Faculty of Engineering, Hokkaido University, Kita-13, Nishi-8, Kita-ku, Sapporo 060-8628 Japan; 3 Center for Crystal Science and Technology, University of Yamanashi, 7-32 Miyamae, Kofu 400-8511 Japan; 4 Department of Physical Science, Hiroshima University, 1-3-1 Kagamiyama, Higashihiroshima, Hiroshima 739-8526 Japan

## Abstract

BiCh_2_-based compounds (Ch: S, Se) are a new series of layered superconductors, and the mechanisms for the emergence of superconductivity in these materials have not yet been elucidated. In this study, we investigate the relationship between crystal structure and superconducting properties of the BiCh_2_-based superconductor family, specifically, optimally doped Ce_1−*x*_Nd_*x*_O_0.5_F_0.5_BiS_2_ and LaO_0.5_F_0.5_Bi(S_1−*y*_Se_*y*_)_2_. We use powder synchrotron X-ray diffraction to determine the crystal structures. We show that the structure parameter essential for the emergence of bulk superconductivity in both systems is the in-plane chemical pressure, rather than Bi-Ch bond lengths or in-plane Ch-Bi-Ch bond angle. Furthermore, we show that the superconducting transition temperature for all REO_0.5_F_0.5_BiCh_2_ superconductors can be determined from the in-plane chemical pressure.

Most superconductors with a high transition temperature (*T*_c_) possess a layered crystal structure. Typical examples of layered high-*T*_c_ superconductors are Cu-oxide^1^ and Fe-based superconductors^2^; the crystal structures of these superconductors are composed of alternating superconducting layers (CuO_2_ or FeAs layers, respectively) and electrically-insulating spacer layers. The spacer layers are essential for the emergence of low-dimensional electronic states within the superconducting layers, which sometimes result in unconventional pairing mechanisms. One of the attractive features of the layered superconductors is the wide variety of crystal structures. New superconductors can be designed by stacking superconducting layers and various types of spacer layers; the superconducting properties depend on the type of the spacer layer. In fact, many Fe-based superconductors containing various types of spacer layers have been discovered, several of which attained high *T*_c_^2^,^3^,^4^,^5^,^6^,^7^,^8^.

With this wide selection of related layered superconductors having various structures and properties, one can determine which crystal structure parameters are essential for the emergence of superconductivity in a given family of layered superconductors. It was found that *T*_c_ for the Fe-based family can be estimated from a crystal structure parameter, such as the As-Fe-As bond angle^9^ or the anion height from the Fe square lattice (anion = As, P, Se, and Te)^10^. Changes in these parameters strongly affect Fermi surface configurations and pairing symmetry, subsequently affecting the superconducting properties of Fe-based superconductors^11^. In fact, clarification of which crystal structure parameter influences superconducting properties is one of the most important challenges for understanding the mechanisms of superconductivity in a new series of layered superconductors; this also provides a direct strategy to design new layered superconductors with high *T*_c_.

In 2012, we reported layered superconductors composed of alternate stacks of BiS_2_ superconducting layers and various spacer layers^12^,^13^. Band calculations suggested that the parent compound of BiS_2_-based superconductors is an insulator containing Bi^3+^. Superconductivity is induced when electron carriers are doped into the BiS_2_ layers (in Bi-6p orbitals) by element substitution in the spacer layers^12^,^14^. For example, a parent compound REOBiS_2_ becomes a superconductor when O^2−^ in the spacer layers is partially substituted by F^−^ (namely, REO_1−*x*_F_*x*_BiS_2_)^13^. To date, 12 types of parent compounds of BiS_2_-based superconductors have been discovered: REOBiS_2_ (RE = La^13^, Ce^15^, Pr^16^, Nd^17^, Sm^18^, Yb^19^, and Bi^20^,^21^), AFBiS_2_ (A = Sr^22^,^23^, Eu^24^), Bi_6_O_8_S_5_^12^, Bi_3_O_2_S_3_^25^, and Eu_3_F_4_Bi_2_S_4_^26^. Superconductivity was also observed in a BiSe_2_-based compound, LaO_0.5_F_0.5_BiSe_2_^27^. Exploring new these compounds is a growing field of research in condensed matter physics.

As described above, electron-carrier doping is necessary for the emergence of superconductivity in the BiCh_2_ family. Some high-pressure (HP) studies, however, suggest that the crystal structure is also important for the emergence of superconductivity in these materials^28^,^29^,^30^,^31^,^32^. One such study investigated the effect of HP on *T*_c_, in which LaO_0.5_F_0.5_BiS_2_ showed remarkable changes in superconductivity under HP. LaO_0.5_F_0.5_BiS_2_ did not exhibit bulk superconductivity (bulk SC), though it showed a filamentary (weak) superconductivity signal with a *T*_c_ of 2.5 K^13^. With the application of HP, LaO_0.5_F_0.5_BiS_2_ become a bulk superconductor and *T*_c_ drastically increases from 2.5 K to over 10 K^13^,^28^,^29^,^30^,^31^,^32^. *T*_c_ enhancement under HP has also been observed in other REO_0.5_F_0.5_BiS_2_ superconductors^32^. These facts strongly suggest that superconducting properties of BiCh_2_-based superconductors are correlated with changes in crystal structure, which is analogous to findings for the Fe-based family^9^,^10^.

Furthermore, our recent studies concerning the effect of isovalent-substitution on superconductivity suggest that optimization of crystal structure is important for the emergence of bulk SC and the ability to attain a high *T*_c_ in optimally doped REO_0.5_F_0.5_BiCh_2_. We use the example of Ce_1−*x*_Nd_*x*_O_0.5_F_0.5_BiS_2_^33^ for following discussion. Since in this crystal structure, the valence of Ce and Nd are both 3+, electron carriers in these compounds are essentially the same: the formal valence of Bi is 2.5+. However, bulk SC is induced by the systematic substitution of Ce by Nd, and *T*_c_ increases with increasing Nd concentration (*x*) as shown in Fig. 1a. The emergence of superconductivity in this material was explained by uniaxial lattice shrinkage along the *a*-axis and optimization of the lattice shrinkage ratio, *c*/*a*^33^. Another example of isovalent-substitution systems is LaO_0.5_F_0.5_Bi(S_1−*y*_Se_*y*_)_2_^34^. In this material, the S^2−^ site within the superconducting layers is systematically substituted by Se^2−^. Therefore, the formal valence of Bi (2.5+) should not change with Se substitution. Bulk SC is induced by Se substitution, and *T*_c_ increases with increasing Se concentration (*y*) as shown in Fig. 1b. Se substitution enhances the metallic conductivity of this system and induces bulk SC through lattice volume expansion^34^.

These experimental results obtained from HP studies on REO_0.5_F_0.5_BiS_2_^13^,^28^,^29^,^30^,^31^,^32^ and isovalent-substitution studies on Ce_1−*x*_Nd_*x*_O_0.5_F_0.5_BiS_2_ and LaO_0.5_F_0.5_Bi(S_1−*y*_Se_*y*_)_2_^33^,^34^, confirm that superconducting properties of the BiCh_2_ family are influenced by not only electron carrier concentration, but also crystal structure. Although previous studies suggest the importance of in-plane Bi-S distance^18^ and/or in-plane S-Bi-S angle^35^ on superconductivity, the universal relationship between crystal structure and superconductivity (and its *T*_c_) in the BiCh_2_ family has not been clarified. In this study, we aim to determine a crystal structure parameter which universally explains the emergence of superconductivity and evolution of *T*_c_ in the BiCh_2_ family. We studied two isovalent-substitution systems, Ce_1−*x*_Nd_*x*_O_0.5_F_0.5_BiS_2_ and LaO_0.5_F_0.5_Bi(S_1−*y*_Se_*y*_)_2_, which exhibit similar superconductivity phase diagrams as shown in Fig. 1. To determine changes in crystal structure parameters and their relationship to superconductivity, we performed powder synchrotron X-ray diffraction (XRD) and Rietveld refinement for these two systems.

Here, we show that the structure parameter essential for the emergence of bulk SC in the BiCh_2_-based family is the in-plane chemical pressure, but not the Bi-Ch bond lengths or in-plane Ch-Bi-Ch angle. Furthermore, we show that *T*_c_ for REO_0.5_F_0.5_BiCh_2_ superconductors can be universally determined from the in-plane chemical pressure. We believe that the important role of in-plane chemical pressure in the evolution of superconductivity in REO_0.5_F_0.5_BiCh_2_ demonstrated here will be useful for designing new Bi-Ch-based layered superconductors with high *T*_c_.

## Results

### Evolution of crystal structure parameters

**Figure 1:**
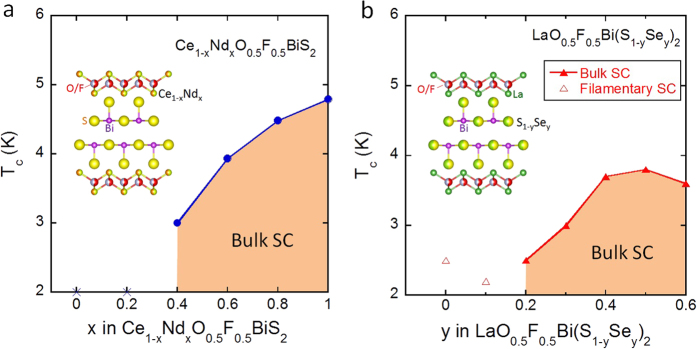
Superconductivity phase diagrams of Ce_1−*x*_Nd_*x*_O_0.5_F_0.5_BiS_2_ and LaO_0.5_F_0.5_Bi(S_1−*y*_Se_*y*_)_2_. (**a**) Superconductivity phase diagrams of Ce_1−*x*_Nd_*x*_O_0.5_F_0.5_BiS_2_. For *x* = 0 and 0.2, superconducting transition is not observed at *T* > 2 K. For 0.4 ≤ *x* ≤ 1, superconducting transition with a large shielding signal, with which we could regard the samples as a bulk superconductor (Bulk SC), is observed. *T*_c_ increases with increasing *x*. The inset figure shows a schematic of crystal structure of Ce_1−*x*_Nd_*x*_O_0.5_F_0.5_BiS_2_. (**b**) Superconductivity phase diagrams of LaO_0.5_F_0.5_Bi(S_1−*y*_Se_*y*_)_2_. For *y* = 0 and 0.1, superconducting transition is observed but their shielding signals are very small as a bulk superconductor (Filamentary SC). For *y *≥ 0.2, superconducting transition with a large shielding signal is observed (Bulk SC). *T*_c_ increases with increasing *y* up to *y* = 0.5. The inset figure shows a schematic image of crystal structure of LaO_0.5_F_0.5_Bi(S_1−*y*_Se_*y*_)_2_.

**Figure 2:**
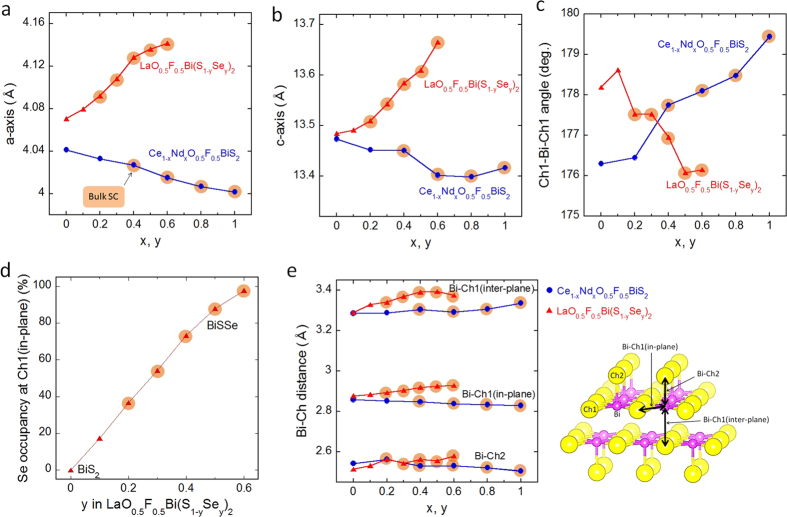
Crystal structure parameters for Ce_1−*x*_Nd_*x*_O_0.5_F_0.5_BiS_2_ and LaO_0.5_F_0.5_Bi(S_1−*y*_Se_*y*_)_2_. (**a**) Lattice constant of *a* for Ce_1−*x*_Nd_*x*_O_0.5_F_0.5_BiS_2_ and LaO_0.5_F_0.5_Bi(S_1−*y*_Se_*y*_)_2_ is plotted as a function of *x* (or *y*). Data points for the samples showing Bulk SC in Fig. 1 are highlighted with orange filled circles in (a–e). (**b**) Lattice constant of *c* for Ce_1−*x*_Nd_*x*_O_0.5_F_0.5_BiS_2_ and LaO_0.5_F_0.5_Bi(S_1−*y*_Se_*y*_)_2_ is plotted as a function of *x* (or *y*). (**c**) Evolution of Ch1-Bi-Ch1 angle for Ce_1−*x*_Nd_*x*_O_0.5_F_0.5_BiS_2_ and LaO_0.5_F_0.5_Bi(S_1−*y*_Se_*y*_)_2_ as a function of *x* (or *y*). (**d**) Se occupancy at the in-plane Ch1 site in LaO_0.5_F_0.5_Bi(S_1−*y*_Se_*y*_)_2_ as a function *y*. (**e**) Evolution of three different Bi-Ch distances in Ce_1−*x*_Nd_*x*_O_0.5_F_0.5_BiS_2_ and LaO_0.5_F_0.5_Bi(S_1−*y*_Se_*y*_)_2_ as a function of *x* (or *y*). The right image describes the Bi, Ch1 and Ch2 sites, and three Bi-Ch distances: Bi-Ch1 (in-plane), Bi-Ch1 (inter-plane), and Bi-Ch2.

We performed powder synchrotron XRD and Rietveld refinement analysis for Ce_1−*x*_Nd_*x*_O_0.5_F_0.5_BiS_2_ and LaO_0.5_F_0.5_Bi(S_1−*y*_Se_*y*_)_2_. Typical Rietveld refinement profiles for *x* = 0.6 and *y* = 0.5 are displayed in the supplementary information (Figure S1). Although minute impurity phases of the RE fluorides were detected, the structures of the main phase was refined using a tetragonal *P*4/*nmm* space group. The crystal structure parameters are plotted as a function of *x* (or *y*) in Fig. 2; the crystal structure data are listed in the supplementary information (Table S1). In Fig. 2, the data points for the samples showing bulk SC are highlighted with orange-filled circles. From Fig. 1, we note that Ce_1−*x*_Nd_*x*_O_0.5_F_0.5_BiS_2_ and LaO_0.5_F_0.5_Bi(S_1−*y*_Se_*y*_)_2_ exhibit similar superconductivity phase diagrams as a function of *x* or *y*. Therefore, if a crystal structure parameter for these two series changes similarly, the parameter should be considered essential for the emergence of superconductivity in these series.

Figure 2a,b show the *x* (or *y*) dependences of the lattice constants *a* and *c* for Ce_1−*x*_Nd_*x*_O_0.5_F_0.5_BiS_2_ and LaO_0.5_F_0.5_Bi(S_1−*y*_Se_*y*_)_2_, respectively. In Ce_1−*x*_Nd_*x*_O_0.5_F_0.5_BiS_2_, both lattice constants decrease with increasing *x* due to increasing concentration of Nd^3+^ (with an ionic radius of 112 pm, assuming a coordination number of 8), which is smaller than Ce^3+^ (with an ionic radius of 114 pm). Conversely, in LaO_0.5_F_0.5_Bi(S_1−*y*_Se_*y*_)_2_, both lattice constants increase with increasing *y* due to the increase of Se^2−^ (with an ionic radius of 198 pm, assuming a coordination number of 6) concentration, which is larger than S^2−^ (with an ionic radius of 184 pm). These contrasting changes in lattice constants suggest that the evolution of superconductivity in these two series cannot be explained by simple lattice contraction or expansion.

Figure 2c shows the *x* (or *y*) dependences of Ch1-Bi-Ch1 angle for Ce_1−*x*_Nd_*x*_O_0.5_F_0.5_BiS_2_ and LaO_0.5_F_0.5_Bi(S_1−*y*_Se_*y*_)_2_. The Ch1-Bi-Ch1 angle is an indicator of the flatness of the Bi-Ch1 plane. Since electrons in the hybridized Bi-6p/Ch-p orbitals (S-3p or Se-4p) are essential for the emergence of superconductivity in BiCh_2_-based superconductors, a flatter Bi-Ch1 plane should facilitate the emergence of bulk SC with high *T*_c_. A study of the crystal structure of CeO_1−*x*_F_*x*_BiS_2_ with different F concentrations demonstrated that a flatter Bi-S1 plane resulted in higher superconducting properties^36^. In Ce_1−*x*_Nd_*x*_O_0.5_F_0.5_BiS_2_, the Ch1-Bi-Ch1 angle approaches 180° with increasing *x*, which indicates that the Bi-S1 plane becomes flatter with Nd substitution, and superconductivity is induced. In contrast, in LaO_0.5_F_0.5_Bi(S_1−*y*_Se_*y*_)_2_, the dependence of the Ch1-Bi-Ch1 angle on Se concentration decreases with increasing *y*, leading to distortion of the Bi-Ch1 plane. Therefore, the Ch1-Bi-Ch1 angle (i.e. the flatness of the Bi-Ch1 plane) cannot explain the evolution of superconductivity in these BiCh_2_-based superconductors. The contrasting changes in the in-plane structure (flatness) may be due to the difference in Ch^2−^ ions at the Ch1 site. Figure 2d shows the *y* dependence of Se occupancy at the Ch1 site, indicating that Se ions selectively occupy the in-plane Ch1 site. At *x* = 0.5, approximately 90% of Ch1 sites are occupied with Se. Recently, a similar site selectivity of Se in LaO_1−*x*_F_*x*_BiSSe single crystals was reported^37^. The preferential occupation of Se^2−^, which has a larger ionic radius, in the two-dimensional Bi-Ch1 plane may cause distortion.

Figure 2e shows the *x* (or *y*) dependences of the three different Bi-Ch distances. Figure 2e (right) demonstrates the BiCh_2_ layer structure, where one Bi ion is coordinated by six Ch ions. The shortest Bi-Ch distance is the Bi-Ch2 distance along the *c*-axis. The Bi-Ch2 distance in Ce_1−*x*_Nd_*x*_O_0.5_F_0.5_BiS_2_ decreases slightly with increasing *x*. In contrast, the Bi-Ch2 distance in LaO_0.5_F_0.5_Bi(S_1−*y*_Se_*y*_)_2_ increases with increasing *y*. Therefore, this indicates that Bi-Ch2 distance cannot be correlated to the evolution of superconductivity within these two series. The Bi-Ch1 (in-plane) distance in Ce_1−*x*_Nd_*x*_O_0.5_F_0.5_BiS_2_ decreases with increasing *x*. In contrast, that in LaO_0.5_F_0.5_Bi(S_1−*y*_Se_*y*_)_2_ increases with increasing *y*. Thus, the in-plane Bi-Ch1 distance cannot explain the evolution of superconductivity. The longest Bi-Ch distance is Bi-Ch1 (inter-plane), which roughly corresponds to the inter-layer distance of two BiCh_2_ layers. The Bi-Ch1 (inter-plane) distance in Ce_1−*x*_Nd_*x*_O_0.5_F_0.5_BiS_2_ does not change much upon Nd substitution, while that in LaO_0.5_F_0.5_Bi(S_1−*y*_Se_*y*_)_2_ shows a noticeable increase with increasing *y*. Therefore, the Bi-Ch1 (inter-plane) distance cannot typically explain the evolution of superconductivity.

Although we expected the Ch1-Bi-Ch1 angle or one of the Bi-Ch distances to exhibit similar behaviour in Ce_1−*x*_Nd_*x*_O_0.5_F_0.5_BiS_2_ and LaO_0.5_F_0.5_Bi(S_1−*y*_Se_*y*_)_2_, we could not identify any clear correlation between them. However, the assumption that the in-plane Bi-Ch1 distance should, to a certain extent, correlate with the evolution of superconductivity stems from the facts that the superconductivity is induced in the Bi-Ch1 plane, and superconducting properties change remarkably with change in the crystal structure without altering the electron carrier concentration (F concentration). Therefore, we introduce the concept of *in-plane chemical pressure* to discuss the relationship between in-plane structure and the evolution of superconductivity in Ce_1−*x*_Nd_*x*_O_0.5_F_0.5_BiS_2_, LaO_0.5_F_0.5_Bi(S_1−*y*_Se_*y*_)_2_, and other REO_0.5_F_0.5_BiCh_2_ superconductors.

### Influence of in-plane chemical pressure on superconductivity

**Figure 3:**
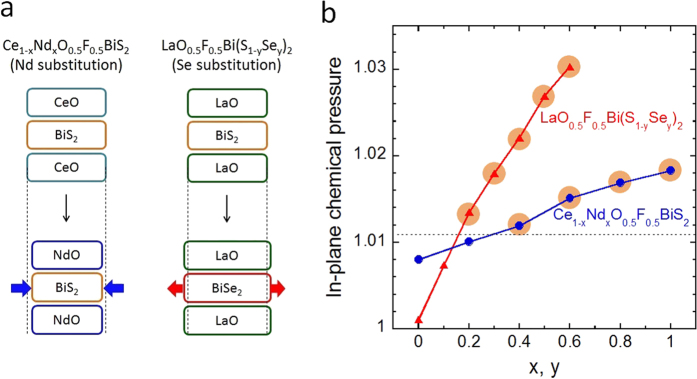
Influence of in-plane chemical pressure to crystal structure and superconductivity in Ce_1−*x*_Nd_*x*_O_0.5_F_0.5_BiS_2_ and LaO_0.5_F_0.5_Bi(S_1−*y*_Se_*y*_)_2_. (**a**) Schematics of changes in crystal structure with increasing in-plane chemical pressure in Ce_1−*x*_Nd_*x*_O_0.5_F_0.5_BiS_2_ and LaO_0.5_F_0.5_Bi(S_1−*y*_Se_*y*_)_2_. In Ce_1−*x*_Nd_*x*_O_0.5_F_0.5_BiS_2_, the volume of spacer layer decreases with increasing Nd concentration (*x*), and Bi-S plane is compressed; hence, in-plane chemical pressure is enhanced. In LaO_0.5_F_0.5_Bi(S_1−*y*_Se_*y*_)_2_, the volume of superconducting Bi-Ch1 layer increases with increasing Se concentration (*y*). However, the expansion of Bi-Ch1 plane is smaller than that expected from the ionic radii of S^2−^ and Se^2−^ because the composition of the spacer layer (LaO layer) remains constant; hence, in-plane chemical pressure is enhanced as well as in Ce_1−*x*_Nd_*x*_O_0.5_F_0.5_BiS_2_. (**b**) In-plane chemical pressure of Ce_1−*x*_Nd_*x*_O_0.5_F_0.5_BiS_2_ and LaO_0.5_F_0.5_Bi(S_1−*y*_Se_*y*_)_2_, calculated using equation (1), are plotted as a function of *x* (or *y*). In both systems, bulk SC is induced with increasing chemical pressure. The dashed line at an in-plane chemical pressure of ~1.011 is an estimated boundary of Bulk-SC and non-SC regions.

Figure 3a shows schematics of compression or expansion of the Bi-Ch plane caused by Nd or Se substitution. In Ce_1−*x*_Nd_*x*_O_0.5_F_0.5_BiS_2_, Bi-Ch1 planes are compressed owing to a decrease in the volume of spacer layers with increasing Nd concentration. The compression of the Bi-Ch1 plane results in an enhancement of the packing density of Bi^2.5+^ and S^2−^ ions within the superconducting plane: this is the so-called *in-plane chemical pressure*. In LaO_0.5_F_0.5_Bi(S_1−*y*_Se_*y*_)_2_, the in-plane Bi-Ch1 distance increases with increasing occupancy of Se at the Ch1 site. However, the increase of the in-plane Bi-Ch1 distance is smaller than that expected from the difference in the ionic radii of S^2−^ and Se^2−^ because the composition of the spacer layer (LaO) remains constant in LaO_0.5_F_0.5_Bi(S_1−*y*_Se_*y*_)_2_. Therefore, the packing density of Bi^2.5+^ and Ch^2−^ ions in the superconducting plane is enhanced. This situation is similar to the enhancement of in-plane chemical pressure in Ce_1−*x*_Nd_*x*_O_0.5_F_0.5_BiS_2_. In order to compare the magnitude of in-plane chemical pressure in the two series, we define in-plane chemical pressure using equation (1).


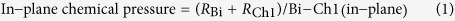


*R*_Bi_ is the ionic radius of Bi^2.5+^. Here, we assume that the ionic radius of Bi^2.5+^ is 104.19 pm, which is obtained from the average of the six Bi-S bonds (four in-plane Bi-S1 bonds, one inter-plane Bi-S1 bond, and one Bi-S2 bond) determined from the structure analysis of a single crystal of LaO_0.54_F_0.46_BiS_2_^35^. *R*_Ch1_ is the ionic radius of the chalcogen at the Ch1 site: 184 and 198 pm for S^2−^ and Se^2−^, respectively. In the case of LaO_0.5_F_0.5_Bi(S_1−*y*_Se_*y*_)_2_, we calculated an average value for *R*_Ch1_ using the occupancy of Se at the Ch1 site. The Bi-Ch1 (in-plane) distances were obtained from the Rietveld refinement (Fig. 2e). We note that the chemical pressure derived from ionic radii is a simplified estimation, and it cannot describe the exact orbital overlap between Bi and Ch. Nevertheless, the estimation appears very useful to discuss the relationship between crystal structure and superconductive properties of these systems.

The calculated in-plane chemical pressure is plotted as a function of *x* (or *y*) in Fig. 3b. For both systems, the in-plane chemical pressure increases with increasing *x* (or *y*). Surprisingly, the chemical pressure at which bulk SC is induced is similar (above ~1.011) in both Ce_1−*x*_Nd_*x*_O_0.5_F_0.5_BiS_2_ and LaO_0.5_F_0.5_Bi(S_1−*y*_Se_*y*_)_2_. Based on these experimental facts, we suggest that the emergence of bulk SC in these systems can be explained by the increase of in-plane chemical pressure. Enhancement of the in-plane chemical pressure would enhance the overlap of Bi-6p and Ch-p orbitals, which would result in an increase of the metallic conductivity and induce bulk SC in the REO_0.5_F_0.5_BiCh_2_ family.

## Discussion

**Figure 4:**
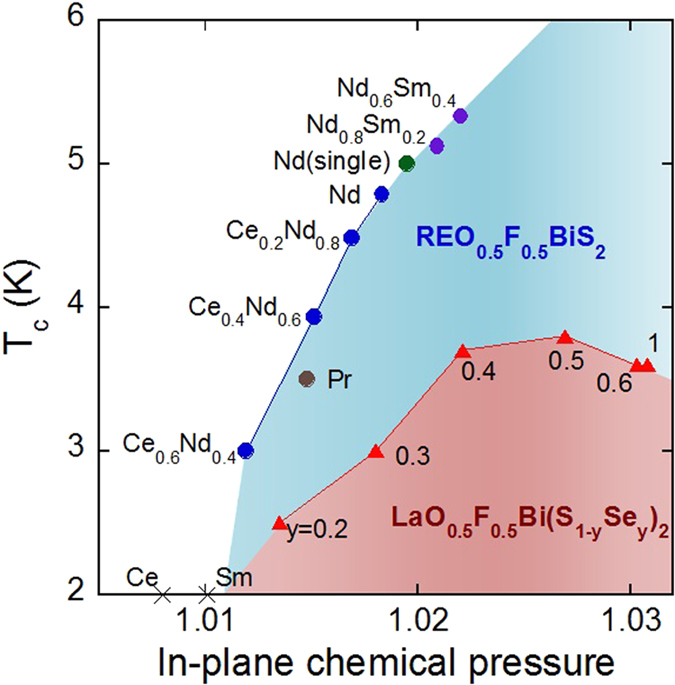
Relationship between *T*_c_ and degree of in-plane chemical pressure in REO_0.5_F_0.5_BiCh_2_. The data of *T*_c_ and the in-plane chemical pressure of Ce_1−*x*_Nd_*x*_O_0.5_F_0.5_BiS_2_ and LaO_0.5_F_0.5_Bi(S_1−*y*_Se_*y*_)_2_ are plotted with those of Nd_0.8_Sm_0.2_O_0.5_F_0.5_BiS_2_ and Nd_0.6_Sm_0.4_O_0.5_F_0.5_BiS_2_ (from this study), PrO_0.5_F_0.5_BiS_2_^16^, and single crystals of NdO_0.5_F_0.5_BiS_2_^38^, SmO_0.5_F_0.5_BiS_2_^18^, and LaO_0.5_F_0.5_BiSe_2_ (*y* = 1)^39^. The curves for REO_0.5_F_0.5_BiS_2_ (blue) and LaO_0.5_F_0.5_Bi(S_1−*y*_Se_*y*_)_2_ (red) lie on different regions.

Figure 4 shows the plot of *T*_c_ for Ce_1−*x*_Nd_*x*_O_0.5_F_0.5_BiS_2_ and LaO_0.5_F_0.5_Bi(S_1−*y*_Se_*y*_)_2_ as a function of in-plane chemical pressure. To clarify a general tendency of superconductivity in the REO_0.5_F_0.5_BiCh_2_ family, we added data points of Nd_0.8_Sm_0.2_O_0.5_F_0.5_BiS_2_ and Nd_0.6_Sm_0.4_O_0.5_F_0.5_BiS_2_ (from this study), PrO_0.5_F_0.5_BiS_2_^16^, and single crystals of NdO_0.5_F_0.5_BiS_2_^38^, SmO_0.5_F_0.5_BiS_2_^18^, and LaO_0.5_F_0.5_BiSe_2_ (*y* = 1)^39^. Interestingly, data points for all the REO_0.5_F_0.5_BiS_2_-type compounds are located on a single curve bounding the blue region. Notably, the data points for CeO_0.5_F_0.5_BiS_2_ and SmO_0.5_F_0.5_BiS_2_, which do not exhibit superconductivity, are located to the left of the boundary (in-plane chemical pressure <1.011). These facts suggest that the emergence of superconductivity and the *T*_c_ of REO_0.5_F_0.5_BiS_2_-type materials simply depend on the magnitude of in-plane chemical pressure. According to the Bardeen-Cooper-Schrieffer (BCS) theory involving electron-phonon mechanisms^40^, the enhancement of *T*_c_ can be explained by an increase of phonon frequency and/or an enhancement of the density of states at the Fermi level. Usually, in metals, an increase in the orbital overlap should decrease the density of states because of an increase of bandwidth. Therefore, the increase in the density of state cannot simply explain the enhancement of *T*_c_ in this series. Although phonon frequency may be enhanced with increasing in-plane chemical pressure, further experimental and theoretical investigations are needed to elucidate the mechanisms of the enhancement of *T*_c_ with increasing in-plane chemical pressure.

A further inspection of Fig. 4 reveals that *T*_c_ for LaO_0.5_F_0.5_Bi(S_1−*y*_Se_*y*_)_2_ and LaOBiSe_2_, shown by the red region, are clearly lower than those for the REO_0.5_F_0.5_BiS_2_-type series. An important observation is that *T*_c_ vs. in-plane chemical pressure plots for REO_0.5_F_0.5_BiS_2_ and LaO_0.5_F_0.5_Bi(S_1−*y*_Se_*y*_)_2_ lie in different regions. For the LaO_0.5_F_0.5_Bi(S_1−*y*_Se_*y*_)_2_ compounds, in-plane chemical pressure is relatively higher than that in REO_0.5_F_0.5_BiS_2_, but their *T*_c_’s are noticeably lower than those of REO_0.5_F_0.5_BiS_2_. As described above, Se preferably occupies the in-plane Ch1 site. Therefore, it can be considered that superconductivity in LaO_0.5_F_0.5_Bi(S_1−*y*_Se_*y*_)_2_ is induced in the Bi-(S,Se) or Bi-Se plane. From the perspective of the BCS theory, the lower *T*_c_ in the Bi-Se plane than in the Bi-S plane can be explained by a lower phonon frequency in the firmer owing to the larger atomic number of Se. To confirm the above assumption and the mechanisms of superconductivity in the BiCh_2_-based superconductor family, further studies of other REO_0.5_F_0.5_BiSe_2_ superconductors, such as CeO_0.5_F_0.5_BiSe_2_ or NdO_0.5_F_0.5_BiSe_2_, are needed for comparison. From Fig. 4, we conclude that a higher *T*_c_ should be obtained for the Bi-S plane with a higher in-plane chemical pressure.

Finally, we briefly discuss the HP phase of LaO_0.5_F_0.5_BiS_2_, which shows the highest *T*_c_ among BiCh_2_-based superconductors^13^,^28^,^29^,^30^,^31^,^32^. T. Tomita *et al.* reported that the crystal structure of LaO_0.5_F_0.5_BiS_2_ above 0.7 GPa was monoclinic^31^. In the monoclinic structure, the Bi-S1 plane is distorted into a zigzag chain with a Bi-S1 distance of 2.72 Å; the zigzag chains are connected with a Bi-S1 distance of 3.03 Å. In addition, we have previously reported the in-plane anisotropy of the upper critical field within the Bi-S1 plane in an HP phase of LaO_0.5_F_0.5_BiS_2_, implying the emergence of quasi-one-dimensional superconducting states in the distorted plane^41^. Calculating the value of chemical pressure by substituting a Bi-S1 distance of 2.72 Å in equation (1) yields chemical pressure of 1.06. In this case, the chemical pressure is not the in-plane chemical pressure but instead is the quasi-one-dimensional chemical pressure. A chemical pressure of 1.06 and *T*_c_ of 10 K roughly lie on the extrapolated *T*_c_-chemical pressure curve for the other REO_0.5_F_0.5_BiS_2_ superconductors shown in Fig. 4. This suggests that the enhancement of quasi-one-dimensional chemical pressure, but not in-plane chemical pressure, is responsible for the evolution of superconductivity in REO_0.5_F_0.5_BiCh_2_ compounds. To further discuss and understand the relationship between chemical pressure and superconductivity in the BiCh_2_ family, investigations of crystal structure and superconducting properties of new BiS_2_-based, BiSe_2_-based and/or BiTe_2_-based compounds with various spacer layers are needed.

In summary, we analysed the crystal structure of the optimally doped BiCh_2_-based superconductors, Ce_1−*x*_Nd_*x*_O_0.5_F_0.5_BiS_2_ and LaO_0.5_F_0.5_Bi(S_1−*y*_Se_*y*_)_2_ using powder synchrotron XRD. We investigated the relationship between the crystal structure and the superconducting properties for these compounds. We found that an enhancement of the in-plane chemical pressure could induce bulk SC in both systems. Furthermore, we revealed that *T*_c_ for the REO_0.5_F_0.5_BiCh_2_ superconductors could be determined by the magnitude of in-plane chemical pressure and type of Ch element that composes the superconducting Bi-Ch planes. In addition, we extended our results to include a monoclinic phase (HP phase) of LaO_0.5_F_0.5_BiS_2_. We suggested that the enhancement of the quasi-one-dimensional chemical pressure within a Bi-Ch chain, but not of the in-plane (two-dimensional) chemical pressure, is required for high *T*_c_ in BiCh_2_-based superconductors.

## Methods

Polycrystalline samples of Ce_1−*x*_Nd_*x*_O_0.5_F_0.5_BiS_2_ and LaO_0.5_F_0.5_Bi(S_1−*y*_Se_*y*_)_2_ used in this study were prepared using solid state reaction. The detailed synthesis procedures were described in previous reports^33^,^34^. Powder synchrotron X-ray powder diffraction measurements were performed at room temperature at the BL02B2 experimental station of SPring-8 (JASRI; Proposal No. 2014B1003, 2014B1071, and 2015A1441). The wavelength of the radiation beam was 0.49542(4) Å. We have performed the Rietveld refinement (RIETAN-FP^42^) for Ce_1−*x*_Nd_*x*_O_0.5_F_0.5_BiS_2_ and LaO_0.5_F_0.5_Bi(S_1−*y*_Se_*y*_)_2_ using a typical structure model of REOBiCh_2_-based superconductor with a tetragonal space group of *P*4/*nmm*^35^,^37^. Contributions from impurity phases of rare-earth fluorides (REF_3_) and/or Bi_2_S_3_ are included in Rietveld refinement. In addition, we have analysed a crystal structure for two related compounds of Nd_0.8_Sm_0.2_O_0.5_F_0.5_BiS_2_ and Nd_0.6_Sm_0.4_O_0.5_F_0.5_BiS_2_ to enrich the discussion part^33^. The obtained crystal structure parameters are summarized in Table S1. The schematic images of crystal structure were drawn using VESTA^43^.

## Additional Information

**How to cite this article**: Mizuguchi, Y. *et al.* In-plane chemical pressure essential for superconductivity in BiCh_2_-based (Ch: S, Se) layered structure. *Sci. Rep.* **5**, 14968; doi: 10.1038/srep14968 (2015).

## Supplementary Material

Supplementary Information
