# Supplementary material for: In-plane chemical pressure essential for superconductivity in BiCh2-based (Ch: S, Se) layered structure
Source: Sci Rep. 2015 Oct 8;5:14968. doi: 10.1038/srep14968 (PMC4597362; doi:10.1038/srep14968)
Supplement: Supplementary Information [file srep14968-s1.pdf]

Supplementary information

In-plane chemical pressure essential for superconductivity in $BiCh_2$-based (Ch: S, Se) layered structure

Yoshikazu Mizuguchi[1]*, Akira Miura[2], Joe Kajitani[1], Takafumi Hiroi[1], Osuke Miura[1], Kiyoharu Tadanaga[2], Nobuhiro Kumada[3], Eisuke Magome[4], Chikako Moriyoshi[4], Yoshihiro Kuroiwa[4]

1. Department of Electrical and Electronic Engineering, Tokyo Metropolitan University, 1-1, Minami-osawa, Hachioji 192-0397, Japan.
2. Faculty of Engineering, Hokkaido University, Kita-13, Nishi-8, Kita-ku, Sapporo 060-8628 Japan.
3. Center for Crystal Science and Technology, University of Yamanashi, 7-32 Miyamae, Kofu 400-8511 Japan.
4. Department of Physical Science, Hiroshima University, 1-3-1 Kagamiyama, Higashihiroshima, Hiroshima 739-8526 Japan.

Table S1. Obtained crystal structure parameters.

Lattice constants *a* and *c*, *z* coordinate, occupancy of Se at Ch1 site (*g*(Se1)), and reliable factor of Rietveld refinement (R_{wp}) for $Ce_{1-x}Nd_xO_{0.5}F_{0.5}BiS_2$, $LaO_{0.5}F_{0.5}Bi(S_{1-y}Se_y)_2$, $Nd_{0.8}Sm_{0.2}O_{0.5}F_{0.5}BiS_2$, and $Nd_{0.6}Sm_{0.4}O_{0.5}F_{0.5}BiS_2$ are listed. Atomic coordinate for the $REO_{0.5}F_{0.5}BiS_2$ series are RE(0, 0.5, *z*), Bi(0, 0.5, *z*), Ch1(0, 0.5, *z*) , Ch2(0, 0.5, *z*), and O/F(0, 0, 0).

Composition	*a* (Å)	*c* (Å)	*z*(RE)	*z*(Bi)	*z*(Ch1)	*z*(Ch2)	g(Se1) (%)	R_{wp} (%)	Impurities
$CeO_{0.5}F_{0.5}BiS_2$	4.04119(7)	13.4736(3)	0.0975(1)	0.6249(1)	0.3810(6)	0.8136(5)	-	5.8	CeF_3(6.8%)
$Ce_{0.8}Nd_{0.2}O_{0.5}F_{0.5}BiS_2$	4.0330(1)	13.4522(6)	0.0976(2)	0.6255(2)	0.3810(8)	0.8160(8)	-	5.8	CeF_3(6.7%)
$Ce_{0.6}Nd_{0.4}O_{0.5}F_{0.5}BiS_2$	4.02710(8)	13.4510(8)	0.0965(1)	0.6249(1)	0.3792(6)	0.8131(5)	-	4.9	CeF_3(5.7%)
$Ce_{0.4}Nd_{0.6}O_{0.5}F_{0.5}BiS_2$	4.01501(9)	13.4023(4)	0.0977(1)	0.6246(1)	0.3789(6)	0.8135(6)	-	4.9	CeF_3(5.3%)
$Ce_{0.2}Nd_{0.8}O_{0.5}F_{0.5}BiS_2$	4.00682(9)	13.3997(4)	0.0969(1)	0.6248(1)	0.3780(7)	0.8130(6)	-	5.9	REF_3(3.6%)
$NdO_{0.5}F_{0.5}BiS_2$	4.0017(1)	13.4166(5)	0.0973(2)	0.6249(2)	0.3762(9)	0.8117(8)	-	7.4	Bi_2S_3(1.5%) + NdF_3(3.5%)
$Nd_{0.8}Sm_{0.2}O_{0.5}F_{0.5}BiS_2$	3.99205(9)	13.4152(4)	0.0956(1)	0.6246(1)	0.3763(8)	0.8084(6)	-	7.6	unknown
$Nd_{0.6}Sm_{0.4}O_{0.5}F_{0.5}BiS_2$	3.9880(2)	13.4407(6)	0.0963(2)	0.6253(2)	0.377(1)	0.8088(8)	-	9.2	unknown
$LaO_{0.5}F_{0.5}BiS_2$	4.07063(6)	13.4848(3)	0.0978(1)	0.6237(1)	0.3797(6)	0.8103(5)	0.0	5.8	Bi_2S_3(1.0%) + LaF_3(5.1%)
$LaO_{0.5}F_{0.5}BiS_{1.8}Se_{0.2}$	4.07966(3)	13.4914(2)	0.09756(9)	0.62469(7)	0.3779(4)	0.8124(4)	17.3(5)	5.4	LaF_3(3.4%)
$LaO_{0.5}F_{0.5}BiS_{1.6}Se_{0.4}$	4.09211(8)	13.5096(3)	0.0971(2)	0.6260(2)	0.3786(5)	0.8159(7)	36.7(8)	5.6	LaF_3(7.0%)
$LaO_{0.5}F_{0.5}BiS_{1.4}Se_{0.6}$	4.10821(4)	13.5433(2)	0.0964(1)	0.62673(9)	0.3779(3)	0.8146(4)	54.2(5)	5.4	LaF_3(3.0%)
$LaO_{0.5}F_{0.5}BiS_{1.2}Se_{0.8}$	4.12832(4)	13.5848(2)	0.0959(1)	0.62769(8)	0.3780(2)	0.8165(4)	73.4(5)	5.5	LaF_3(2.5%)
$LaO_{0.5}F_{0.5}BiSSe$	4.13593(5)	13.6098(2)	0.0953(1)	0.6284(1)	0.3790(2)	0.8163(4)	87.9(5)	5.1	LaF_3(2.5%)
$LaO_{0.5}F_{0.5}BiS_{0.8}Se_{1.2}$	4.14138(7)	13.6656(3)	0.0944(1)	0.6271(1)	0.3801(3)	0.8160(4)	97.9(7)	6.0	unknown

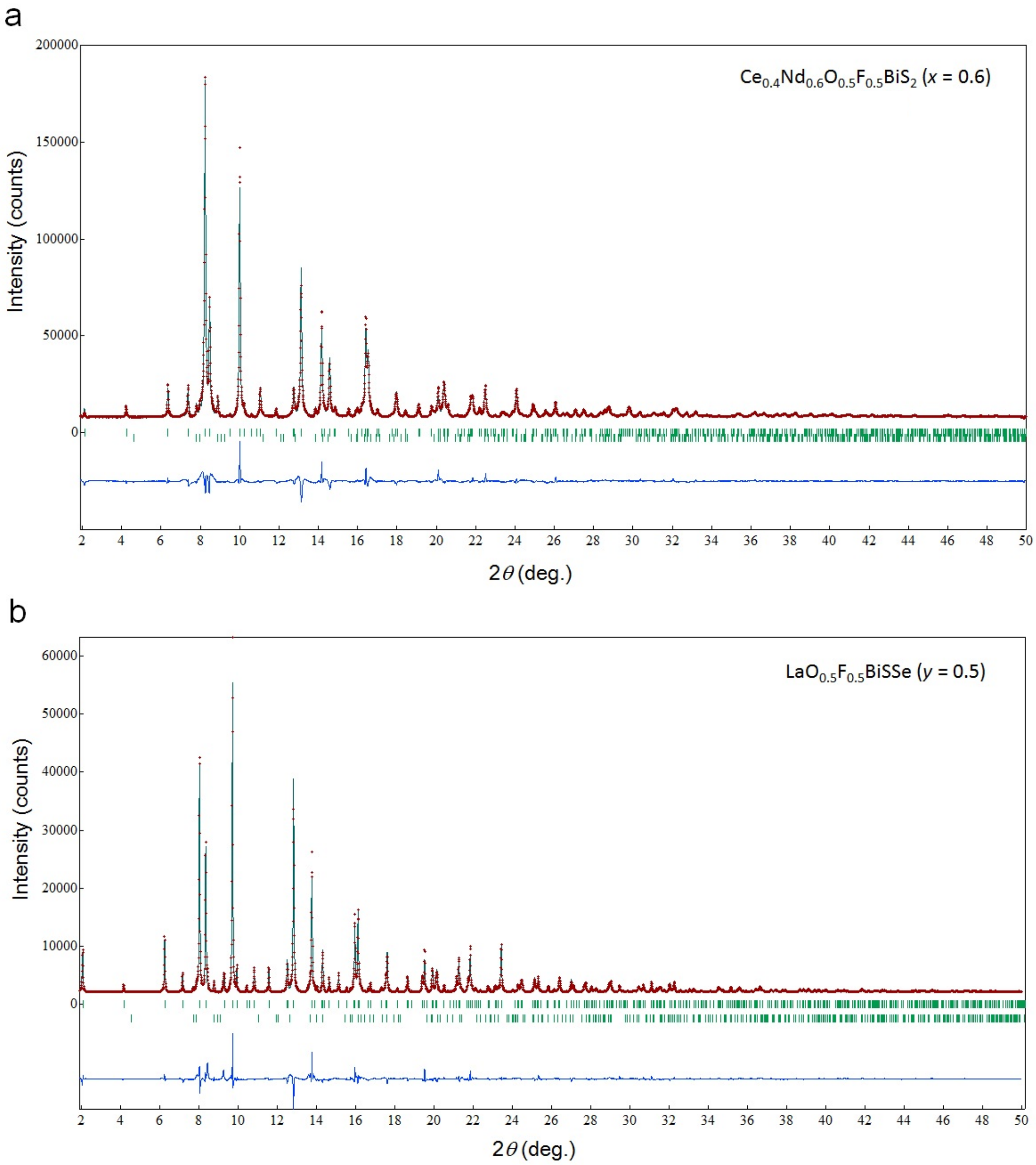

Fig. S1. Typical XRD patterns for $Ce_{1-x}Nd_xO_{0.5}F_{0.5}BiS_2$ and $LaO_{0.5}F_{0.5}Bi(S_{1-y}Se_y)_2$.

a. XRD patterns for $Ce_{0.4}Nd_{0.6}O_{0.5}F_{0.5}BiS_2$ (x = 0.6). The Rietveld refinement was performed including a CeF_3 impurity phase (5.3%). The upper and lower bars indicate calculated peak positions for $Ce_{0.4}Nd_{0.6}O_{0.5}F_{0.5}BiS_2$ and CeF_3, respectively. The reliable factor R_{wp} was 4.9%.

b. XRD patterns for $LaO_{0.5}F_{0.5}BiSSe$ (y = 0.5). The Rietveld refinement was performed including a LaF_3 impurity phase (2.5%). The upper and lower bars indicate calculated peak positions for $LaO_{0.5}F_{0.5}BiSSe$ and LaF_3, respectively. The reliable factor R_{wp} was 5.1%.

[Discussion on the importance of anion-anion interactions in $REO_{0.5}F_{0.5}BiCh_2$]

We have discussed the importance of the anion-anion interactions to understand the physical properties of $REO_{0.5}F_{0.5}BiCh_2$. We have estimated several anion-anion distances for $NdO_{0.5}F_{0.5}BiS_2$ and $LaO_{0.5}F_{0.5}BiSSe$ because the in-plane chemical pressure effects are large enough for the emergence of bulk superconductivity in these compounds.

<$NdO_{0.5}F_{0.5}BiS_2$>

We have estimated S1-S1 distances of $NdO_{0.5}F_{0.5}BiS_2$. The in-plane S1-S1 distance is 4.00 Å, and the S1-S2 distance is 3.79 Å. The inter-plane S1-S1 distance is 4.36 Å. In this material, all the Ch sites are occupied with S^{2-}. Hence, the double of ionic radius of S^{2-} (1.84 Å × 2 = 3.68 Å) could be a scale when discussing the importance of the anion-anion interactions. The shortest S1-S2 distance is 3.79 Å, which is longer than 3.68 Å. Therefore, we consider that the anion-anion interactions would not be significant, and do not largely affect the physical properties and electronic structure as compared to the importance of in-plane Bi-Ch (cation-anion) interaction.

<$LaO_{0.5}F_{0.5}BiSSe$>

The in-plane Ch1-Ch1 distance is 4.13 Å, which is longer than the double of ionic radius of Se^{2-} (1.98 × 2 = 3.96 Å). The inter-plane Ch1-Ch1 distance is 4.40 Å. The Ch1-Ch2 distance of 3.95 Å is the shortest among all the anion-anion distances. This value is comparable to the double of ionic radius of Se^{2-} (1.98 × 2 = 3.96 Å). In real, the Ch2 site is mainly occupied by S^{2-}; hence, we consider that the anion-anion interactions should not greatly affect the physical properties and electronic structure as compared to the case of in-plane Bi-Ch interaction.

Anion-anion interactions would exist in some $BiCh_2$-based materials to a certain extent. However, on the basis of above discussion, we consider that anion-anion interactions should not affect the evolution of superconductivity at least in $Ce_{1-x}Nd_xO_{0.5}F_{0.5}BiS_2$ and $LaO_{0.5}F_{0.5}Bi(S_{1-y}Se_y)_2$. Hence, we conclude that the structure parameter which is the most important for the evolution of superconductivity in these materials is the in-plane chemical pressure as explained in the main text.
